# Successful outpatient conservative management of chyle leak after laparoscopic left hemicolectomy

**DOI:** 10.23938/ASSN.1051

**Published:** 2023-11-22

**Authors:** Laura Rubio López, Silvia Benito Barbero, Javier Páramo Zunzunegui, Teresa Antón Bravo, María Moral González

**Affiliations:** 1 Servicio de Cirugía General y del Aparato Digestivo Hospital Universitario de Móstoles Móstoles Madrid Spain; 2 Universidad Rey Juan Carlos Universidad Rey Juan Carlos Facultad de Ciencias de la Salud Departamento de Especialidades Médicas y Salud Pública Alcorcón Madrid Spain; 3 Servicio de Endocrinología y Nutrición Hospital Universitario de Móstoles Móstoles Madrid España; 4 Universidad Francisco de Vitoria Universidad Francisco de Vitoria Majadahonda Madrid Spain

**Keywords:** Chylous ascites, Food formulated, Enteral nutrition, Colorectal neoplasms, Ascitis quilosa, Alimentos formulados, Nutrición enteral, Cáncer colorrectal

## Abstract

Chyle leak is a pathological extravasation of chyle into the peritoneal cavity after a surgical injury. It is an uncommon complication in colorectal surgery. In most cases, conservative treatment is effective, although it often entails prolonged hospital stays.

We present the case of a 60-year-old female with chyle leak after laparoscopic left hemicolectomy with complete mesocolic excision who underwent successful outpatient conservative management. We found no other cases of successful conservative outpatient treatment in the consulted literature. Adequate outpatient management may provide significant benefits by reducing hospital costs and improving patient’s quality of life, while maintaining the possibility of starting adjuvant treatment if indicated.

## RESUMEN

La ascitis quilosa es una acumulación patológica de quilo en la cavidad peritoneal tras una lesión quirúrgica. Es una complicación infrecuente de la cirugía colorrectal. En la mayoría de los casos, el tratamiento conservador suele ser eficaz, aunque conlleva estancias hospitalarias prolongadas.

Se presenta el caso de una paciente de 60 años que desarrolló ascitis quilosa tras una hemicolectomía izquierda laparoscópica, con escisión completa del mesocolon, que se resolvió con éxito mediante manejo conservador ambulatorio. No se han encontrado casos exitosos de tratamiento conservador ambulatorio en la bibliografía consultada. Un manejo ambulatorio como el propuesto puede aportar importantes beneficios en términos de reducción de costes hospitalarios y mejora de la calidad de vida del paciente, manteniendo la posibilidad de iniciar un tratamiento adyuvante si está indicado.

## INTRODUCTION

Postoperative chyle leak (CL) is an uncommon complication of colorectal surgery related to iatrogenic trauma to lymphatic vessels. The reported incidence of CL is between 1% and 6.6%^1^^-^^5^; however, incidence is increasing due to the rise of more extensive lymphadenectomies^5^^-^^7^. There are different terminologies and diagnostic criteria for CL in the literature. The generally accepted criteria of CL are a leak debit > 200 mL/day and triglyceride levels > 110-200 mg/dL or 2-8 times the plasma concentration^1^^-^^4^.

CL may lead to dehydration, malnutrition, immunosuppression, and longer hospital stays^1^^,^^2^^,^^6^; moreover, it delays adjuvant chemotherapy with the consequent increased risk of disease recurrence due to the potential spread of cancer cells into the CL^5^^,^^8^. Hence the importance of an early diagnosis and treatment initiation.

We present the first described case of successful outpatient treatment of postoperative CL.

## CASE REPORT

**Figure 1:**
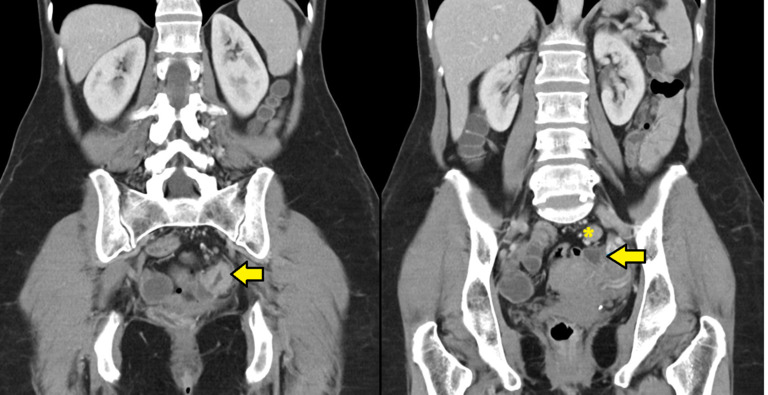
A coronal computed tomography image shows circumferential parietal thickening causing luminal stenosis (arrow). Regional lymph nodes are also observed, the largest (asterisk) of 16 mm for the long axis diameter and suspicious for malignancy.

A 60-year-old female with no relevant medical history nor abdominal symptoms was diagnosed of partially stenosing sigmoid adenocarcinoma after a colonoscopy following a positive faecal occult blood test. A thoracic-abdominopelvic computed tomography revealed suspicious lymphadenopathy (Figura 1).

The patient underwent a laparoscopic left hemicolectomy with complete mesocolic excision by ligation with hem-o-lok’s® (Weck, Teleflex) at the origin of the inferior mesenteric artery and vein and electrodissection with Thunderbeat® (Olympus). The pathological study showed a moderately differentiated sigmoid adenocarcinoma (histological grade 2), with free surgical borders and metastasis in one of the 21 isolated lymph nodes, with pathological stage IIIB (pT3 pN1a).

**Figure 2:**
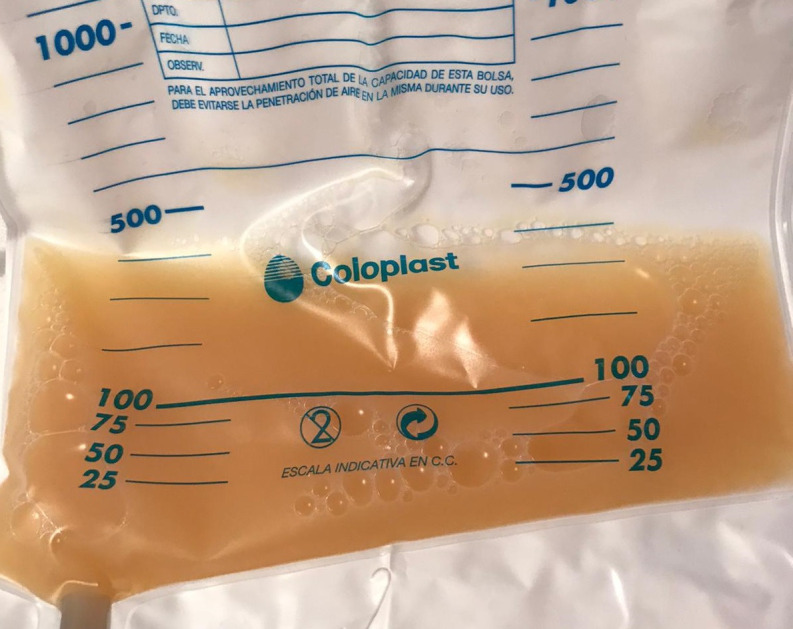
Chyle ascites drainage obtained on Day 2 post-surgery.

After the reintroduction of normal oral diet, on Day 2 post-surgery, the intra-abdominal drain output turned milky and increased in quantity, approximately 500 mL/day (Fig. 2). Clinically, the patient showed no fever and no signs of peritoneal irritation or infection, suggesting anastomotic leakage or other intra-abdominal infection.

The analysis of the drainage fluid showed elevated triglyceride concentration (2,036 mg/dL), protein 3g/dL, lactic dehydrogenase 306 IU/L, amylase 16U/L, and glucose 140 mg/dL. Cytology for malignancy was negative, as well as the microbiological cultures. Postoperative chyle leak was diagnosed due to the lack of other major findings.

A strict fat-free oral diet supplemented with medium-chain triglycerides and subcutaneous octreotide (0.1 mg/8 hours) was started. On Day 4 post-surgery, the patient was offered outpatient management with intra-abdominal drainage and nursing, surgical, and nutritional follow-up. Progressive decrease of the drainage’s debit was observed. One week after the beginning of the conservative treatment, drainage fluid was 250 mL/day debit with a triglyceride concentration of 327 mg/dL. The drainage was clamped with ultrasound monitoring of the intra-abdominal fluid at baseline, and on Day 4 and Day 8 post-clamping. No increase in intra-abdominal fluid was observed; the drainage was removed on Day 13 post-surgery.

Regular oral diet was progressively reintroduced without incidents. Simultaneously, the patient started adjuvant chemotherapy treatment following the schedule. Two years later, the patient remains asymptomatic with no evidence of CL or tumour recurrence.

## DISCUSSION

The diagnosis of postoperative CL is usually achieved rapidly by observing a milky discharge in the intra-abdominal drainage after reintroducing the oral diet^5^, as in our case. However, routine use of prophylactic drainage has no benefits in colorectal surgery^3^. Thus, in the absence of drainage, the patient presents postoperative abdominal distention and pain. To reach a CL diagnosis, an abdominal computed tomography (CT) is needed, in which massive ascites are identified, followed by paracentesis and ascitic fluid analyses^5^.

Many risk factors have been described for the development of CL after colorectal surgery^1^. The most prominent is tumour location, as CL is more frequent after right colectomies^2^^,^^4^^-^^7^, opposite to the case presented here. Other risk factors are advanced age^2^, short operating time^2^^,^^3^, and extensive lymphadenectomy^3^^-^^5^^,^^7^. The incidence of CL has increased due to the rise in the number of complete mesocolic excisions (CME) in colorectal surgery^5^^,^^7^, as in our patient. In addition, the type of used surgical technique and surgical energy devices seem to influence the occurrence of lymphatic leakage. The likelihood of a CL may be higher with the use of electrocautery and ultrasonic devices in comparison to ligatures or hemoclips^2^^,^^10^. In our patient, vascular clips and advanced energy (Thunderbeat^®^) were used.

At the hospital, conservative treatment for postoperative CL provides successful resolution in 66-77% of the cases^1^^,^^11^. This treatment either consists of fasting -with the administration of total parenteral nutrition- or a strict oral diet (high in protein, fat-free, and rich in medium-chain triglycerides, MCTs) to reduce lymphatic flow^1^^,^^2^^,^^9^^,^^10^^,^^12^. In our case, a strict oral fat-free diet supplemented with MCTs was indicated. Several brands of enteral formulas containing MCTs are commercially available or alternatively, liquid oil or capsulated MCTs. The usual adult dose is 50 to 100 mL/day^12^. The administration of somatostatin analogues may be helpful, although evidence remains unclear^1^^,^^2^^,^^12^^,^^13^. Some researchers hold that they can reduce lymphatic production and directly induce the contraction of the lymphatic vessels by acting on lymphatic vessel receptors of the gastrointestinal tract^1^^,^^2^, but the evidence is still limited^5^. We administered subcutaneous (0.1 mg/8 hours) octreotide (a somatostatin analogue for only four days, as no reduction in drainage’s debit was observed.

There is no established length for the conservative therapy. However, if the leak persists after 6-8 weeks, the conservative therapy should be regarded as a failure and surgery considered. The most commonly used surgical treatment is repair under direct vision. For intraoperative location of the lymphatic leak, preoperative administration of high-fat food, lymphangiography, or indocyanine green injection are helpful.^1^^-^^3^^,^^8^^-^^11^^,^^13^. Lymphangiography with lipiodol (a modified poppy seed oil) injection is also a possible therapeutic approach for lymphatic leakage. The mechanism by which it reduces lymphatic leaks has not yet been fully clarified, but it is thought to block lymphatic ducts^15^.

To the best of our knowledge, no cases of conservative outpatient management have been reported to date. Conservative outpatient management prevents extended hospital stays and the need of parenteral nutrition. In addition, it avoids delaying the beginning of adjuvant chemotherapy, an important factor due to generalized concern for increased risk of tumour recurrence following CL^8^.

There is limited literature on the prevention of CL. Giovanni *et al*.^14^ and Ha *et al.*^16^ suggest CL cannot always be prevented due to the inconsistent anatomy of the lymphatic system. However, Baek *et al.*^2^ report that meticulous dissection and the use of vascular clips for vascular ligation, rather than electrosurgical devices, can minimize its occurrence. Agustsdottir *et al.*^10^ suggest the routine implementation of a low-fat diet during the first three postoperative days in patients undergoing extended lymphadenectomy to prevent CL.

In conclusion, CL is an infrequent complication after colorectal surgery. However, because of the rise in the number of CME, the occurrence of CL should be closely monitored after this type of surgery. Although most cases resolve without surgical intervention, it usually entails prolonged hospital stays and a significant increase in the morbidity. We present the first described case of a successful outpatient treatment, demonstrating that outpatient management is possible; moreover, it has economic and quality of life advantages for the patient, and avoids delaying the adjuvant treatment. Further studies are needed to establish an outpatient management protocol. Dissemination of our results may help inform on alternative effective treatment options.
